# The American Dental Association

**Published:** 1865-08

**Authors:** 


### THE AMERICAN DENTAL ASSOCIATION.

The annual meeting of this body was held in Chicago, on the last Tuesday of July.

It was the largest, and we think the most interesting, meeting of this Association ever held. There were about one hundred and twenty members and delegates present, and all were highly interested in the proceedings.

Such arrangements were made as to secure the entire occupancy of the time most advantageously; indeed, the time was too closely occupied for the ease and comfort of the members. Many complained of fatigue at the close of the meeting.

There was perhaps nothing connected with this meeting more gratifying to those present, than the cordial welcome extended to the Association by the medical profession of that city.

Several lectures were delivered by members of the medical profession, before the Association, and also two sumptuous entertainments were given by them.

The papers read and the discussions had upon them, were of a highly entertaining and instructive character. Exhibiting evidence that development and progress are being rapidly made in our profession.

The American Dental Association is established upon a permanent basis, and if the members of the profession fulfill their obligations, it will grow and increase in power and influence, until it will become in many respects *the* power in the profession. The organization is such that there is scarcely room for discord; each and every one should perform fully his part. If any are tardy, apply the spur; if any are officious, let the rein be tightly drawn; let none be unwisely presuming.

The next meeting will be held in Boston, on the last Tuesday of July, 1866, when we confidently expect a much larger meeting than the last. All the local societies throughout the country are in a flourishing and growing condition, and new ones are constantly springing up; and from all these this body is constituted.

